# The Early Stages and Natural History of *Antirrhea Adoptive Porphyrosticta* (Watkins, 1928) in Eastern Ecuador (Lepidoptera: Nymphalidae: Morphinae)

**DOI:** 10.1673/031.009.3001

**Published:** 2009-06-02

**Authors:** Harold F. Greeney, Philip J. DeVries, Carla M. Penz, Rafael B. Granizo-T, Heidi Connahs, John O. Stireman, Thomas R. Walla, Lee A. Dyer

**Affiliations:** ^1^ Yanayacu Biological Station & Center for Creative Studies, Cosanga, Ecuador c/o 721 Foch y Amazonas, Quito, Ecuador; ^2^ University of New Orleans, Department of Biological Sciences, New Orleans, LA 70148; ^3^ University of New Orleans, Department of Biological Sciences, New Orleans, LA 70148; ^4^ Yanayacu Biological Station & Center for Creative Studies, Cosanga, Ecuador c/o 721 Foch y Amazonas, Quito, Ecuador; ^5^ Department of Ecology and Evolutionary Biology, Tulane University, New Orleans, LA 70118; ^6^ Department of Biological Sciences, Wright State University, Dayton, OH 45435, USA; ^7^ Department of Biology, Mesa State College, 1100 North Avenue, Grand Junction, CO 81501, USA; ^8^ Biology Department, University of Nevada, Reno, Nevada

**Keywords:** Bamboo, *Chusquea scandens*, cloud forest, crepuscular, *Antirrhea miltiades*, *Antirrhea philoctetes*, *Antirrhea pterocopha*, Antirrhea weymeri

## Abstract

Here we describe the immature stages and ecological associations of *Antirrhea adoptiva porphyrosticta* Watkins, 1928 (Lepidoptera:Nymphalidae:Morphinae). The cloud forest bamboo, *Chusquea scandens* Kunth (Bambusoidea: Poaceae), serves as the larval food plant for this butterfly in eastern Ecuador, the first hostplant record for *Antirrhea* outside the family Arecaceae. The larvae of *A. adoptiva porphyrosticta* are superficially similar to those of other *Antirrhea* species. We also provide observations on adult and larval behavior. Caterpillars of this butterfly species are parasitized by tachinid flies, as well as by Ichneumonidae and a newly described braconid wasp.

## Introduction

The 20 species of *Antirrhea* Hübner, 1822 (Lepidoptera: Nymphalidae: Morphinae) are distributed from Guatemala to the Amazon Basin and southern Brazil (D'Abrera 1984; Lamas 2004). Like many Neotropical butterfly genera, *Antinhea* reaches its highest diversity on the eastern Andean slope. Adults haunt the deep shade of the forest understory, feeding on rotting fruit and seeming to favor palm fruits, especially those infested with fungi (DeVries et al. 1985; DeVries 1987). While many *Antirrhea* species are diurnal (e.g., DeVries 1987), others appear to be strictly crepuscular (e.g., Heredia and AlvarezLopez 2004). Previously reported larval food plant associations of *Antinhea* are confined to members of the palm family (Arecaceae) (DeVries et al. 1985; DeVries 1986, 1987; Urich and Emmel 1990; Matos 2000; Heredia and Alvarez-Lopez 2004).

*Antinhea adoptiva* (Weymer, 1909), as currently defined, includes two subspecies; nominate *adoptiva* from Colombia and *porphyrosticta* Watkins, 1928 from Ecuador (Lamas 2004). Lamas (2004), however, mentions two additional undescribed subspecies, which would extend the species distribution into Venezuela and Peru. There are no published reports on any aspect of *A.adoptiva* 's biology or behavior. In fact, *A. weymeri* Salazar, Constantino and Lopez, 1998 is the only *Antinhea* species for which a fairly complete life history description is available (Heredia and Alvarez-Lopez 2004). Here we provide observations on the immature stages and natural history of *A. adoptiva porphyrosticta* (*adoptiva* hereafter) from the eastern slope of the northern Ecuadorian Andes.

## Materials and Methods

We made all observations of *A. adoptiva* at the Yanayacu Biological Station and Center for Creative Studies (YBS), situated adjacent to Cabanas San Isidro, 5 km west of Cosanga, Napo Province, eastern Ecuador (00°35.949 S, 77°53.403 W). The area around the station is humid cloud forest, at elevations ranging from 2000–2200 m, in which *Chusquea* Kunth bamboo is a dominant component of the vegetation (see Greeney et al. 2006 and Valencia 1995 for more complete site descriptions). We collected larvae and made observations opportunistically over the course of eight years. We brought all larvae encountered in the field to the biological station, where we reared them in plastic bags or glass jars, providing fresh leaves daily, until they died or eclosed.

## Results

### Egg (Figures 1a, 1b) n = 9; ca. 2 mm wide × 1 mm tall; development time 21–22 days

**Figure 1.**
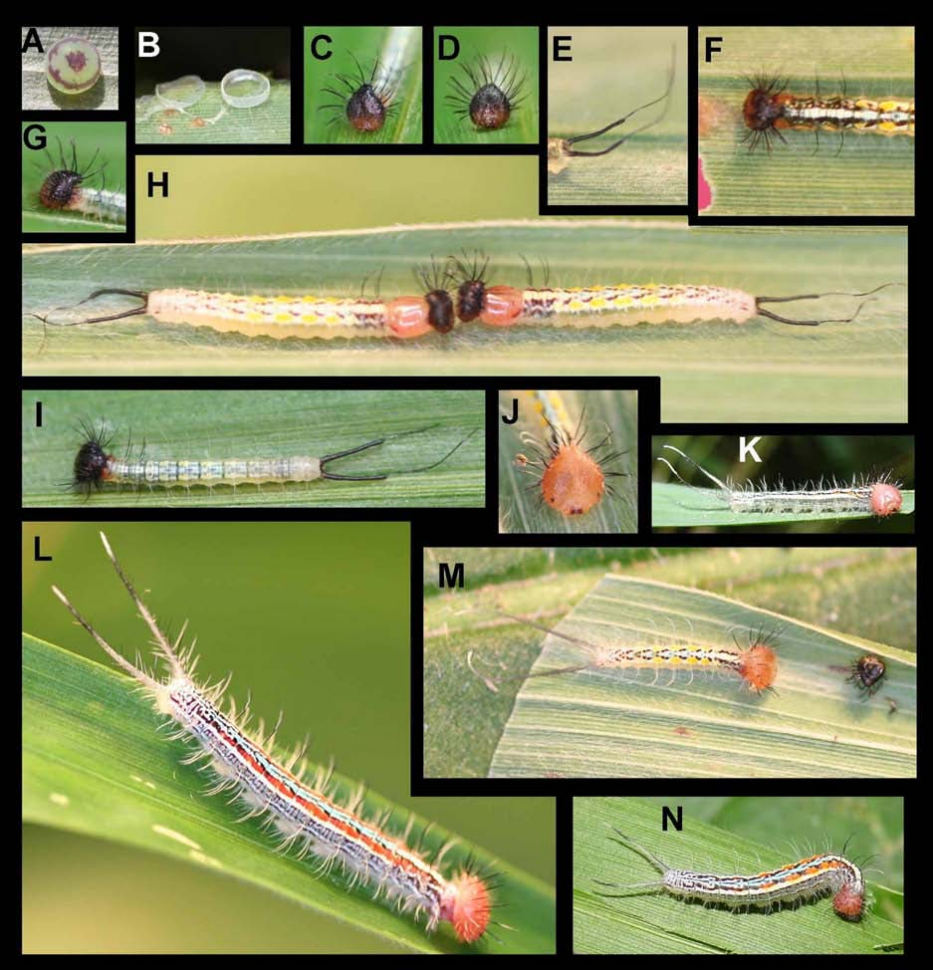
Early stages of *Antirrhea adoptive porphyrosticta* in eastern Ecuador, a) egg soon before hatching; b) remainders of egg shells after hatching; c-d) anterior views of first instar head capsule; e) detail of first instar caudal tails; f) dorsal view of first instar head and thorax; g) lateral view of first instar head; h) premolt first instars; i) first instar; j) head capsule of second instar, k) second instar; I) third instar; m) recently molted second instar; n) third instar.

Egg flattened, hemispherical, smooth, translucent limegreen. With light passing through them from above, eggs are cryptic against the similarly-colored leaves of the food plant. Four days after oviposition, the eggs develop a broad, deep-red circle around the top that is slightly broken at one point (Figure 1a). Later, the area around the micropyle also turns this color, creating a bullseyelike pattern. Clutch size was 4 in one instance and 2 in another. From the first puncture of the chorion, larvae took two to three days to eat their way out of the egg shell, consuming only the top of the egg (Figure 1b).

### First instar (Figures 1c–i) n = 14; body length = 4–11.5 mm; tail = 4 mm; development time = 8–11 days

Head dark brown to black dorsally, fading to light brown basally; mandibles black; shape roughly triangular and projected dorsally into posteriorly curved, slightly bifid scoli; adorned with 30 long, thick, anteriorly-curved setae, arranged laterally in three irregular dorso-ventral rows, these decreasing in size ventrally; each seta slightly compressed basally, becoming less so apically, distally with sparse, short, black barbs along shaft; frontal portion of head with a moderately dense covering of short, soft, pale setae (Figure 1c–d, 1g); prothoracic shield weakly sclerotized, dark brown, broken at midline into two irregular, teardrop-shaped sections extending ventrally into supraspiracular area; T1–T3 bearing short, sparse, pale setae approximately ½ as long as abdominal setae; T2 and T3 with four long, thin, flexible, anteriorly curved black scoli, two arranged subdorsally and two supraspiracularly; body elongate, parallel sided; each segment bearing small, fleshy lateral projections (Figure 1h–i); A1–A9 with 8–12 long, soft, pale setae, pairs arranged dorsally, subdorsally, spiracularly and subspiracularly, posterior segments with lower pairs doubled with two setae arising adjacently; terminus of A10 quadrate, except bearing two long, thin tails, these widely separated at their bases; each tail cylindrical, tapering distally to a sharp constriction from which arises a single, soft, black seta; tails otherwise devoid of setae except for one long, black seta projecting laterally at base (Figure 1e, 1h–i); body ground color transparent-white with dark, purplegreen gut contents often visible anteriorly, caudal tails and dorsal A10 grey, tails darkening to black within two days of hatching.

Early in the instar, patterning on the body is weak (Figure 1i), with the strongest markings being a bright red cast to the prothorax, as well as eight pale yellow dots, one on either side of the midline on A2–A5. Otherwise, the abdominal dorsum is marked with pale white patches forming a faint checkering pattern, extending anteriorly onto the thorax as a broad mid-dorsal stripe. Later in the instar (Figure 1f, h) this pattern becomes more pronounced and the areas between these markings darken to various shades of green, blue, and maroon. The pattern is complex, but is characterized by contrasting dark subdorsal areas on the thorax and a series of dark chevrons on the dorsum from A1–A7. The paler mid-dorsal stripe varies from pale blue to white along its length. Prior to molting (Figure 1h), the prothorax becomes swollen and orange (exposing the color of the next instars' head capsule). When touched with a probe, larvae lift their thoracic legs and A10 prolegs from the substrate and flail with their tails and head. Larvae attempted to hook the probe with their cephalic scoli and violently fling it away.

### Second instar (Figures 1j–k, 1m) n = 12; body length = to 15 mm; tail = 5.5 mm; development time = 14–15 days

Similar to fifth instar, but color pattern not as distinct.

### Third instar (Figure 1l–n) n = 21; body length = to 23 mm; tail = 7.0 mm; development time = 14–17 days

Similar to second through fifth instars; body pattern more clearly defined than in previous instar.

### Fourth instar (Figures 2a, 2d) n = 17; body length = to 36 mm; tail = 10 mm; development time = 16–19 days

Color and pattern nearly identical to fifth instar.

### Fifth instar (Figures 2b–c, 2e–f, 3a–c, 3h) n = 27; body length = to 45 mm; tail = 12 mm; development time = 20–26 days

**Figure 2.**
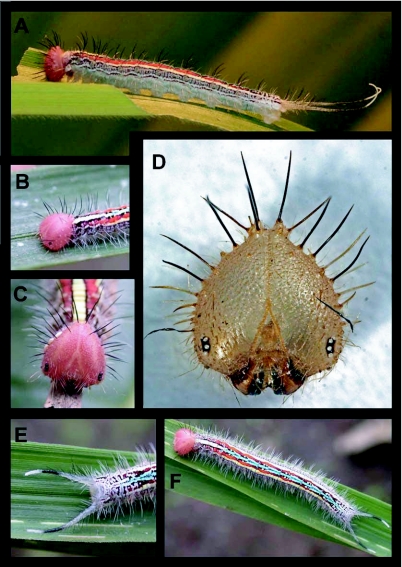
Early stages of *Antirrhea adoptiva porphyrosticta* in eastern Ecuador, a) fourth instar; b) lateral view of fifth instar head; c) anterior view of fifth instar head showing tachinid eggs glued to the left; d) shed head capsule of fourth instar; e) detail of caudal tails of fifth instar; f) mature fifth instar.

**Figure 3.**
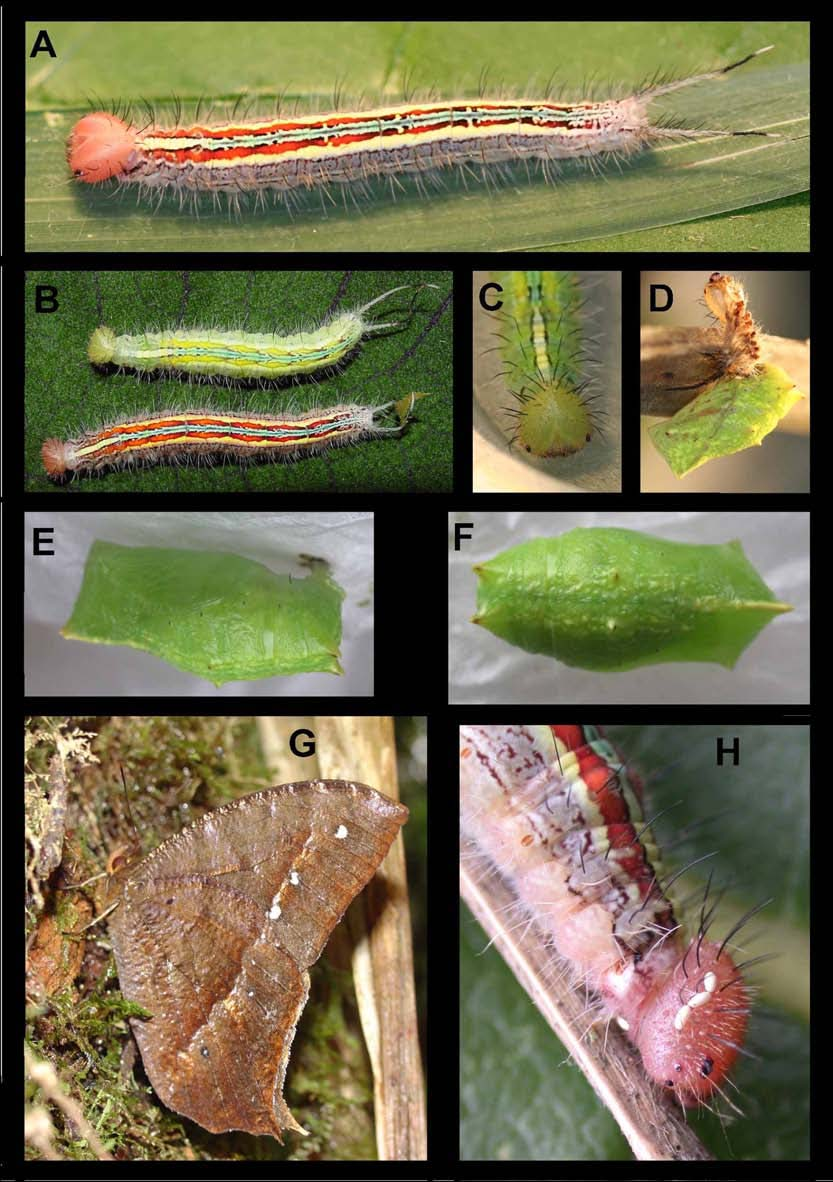
Early stages of *Antirrhea adoptive porphyrosticta* in eastern Ecuador, a) mature fifth instar; b) prepupal (top) and mature fifth instars; c) anterior view of head of prepupal larva; d-f) pupa; g) adult; h) lateral view of fifth instar showing tachind eggs attached to side of head (3 eggs) and the venter of T1 (1 egg).

Head capsule roundly triangular, narrowing apically with epicranium extended into two short, rounded, posteriorly oriented scoli; entirely dull pink to orange colored, mandibles dark pink to brown, kidney-shaped, dark brown areas at stemmata form small eye-spot markings; entire head with short, sparse, soft, pale setae except anteriobasally below ommatidia, setae shorter anteriorly; 4 to 6 longer, laterally and anteriorly projecting, pale setae subtend to stemmata and surrounding mouthparts; a sparse fringe of 10–12 long, stiff, black setae extend laterally from stemmata dorsally to epicranium (Figures 2b–c, 3h); body elongate, roughly trapezoidal in cross section, parallel-sided with T1 slightly constricted and posterior abdominal segments narrowing slightly (Figures 2f, 3a); A10 with a pair of long, soft, pincer-like caudal tails (Figure 2e); body ground color chalky white to light pink, irregular rust-colored markings laterally giving a dirty appearance; mid-dorsal stripe bright canary yellow from T1 to A1 grading to bright aquamarine blue beginning on A2 and extending to A9; middorsal stripe fading and becoming less defined posteriorly; T2 to A5 subdorsally, bordering middorsal stripe, with many small black dots or dashes, from A6 to A9 becoming sparser; A1 to A8 with narrow, bright orange-yellow, irregular subdorsal to supraspiracular stripe, fading to yellow-green posteriorly and thinly bordered with dull olive anteriorly, this stripe subtended by diffuse bright yellow wash, especially on A4 to A6; caudal tails clear to whitish with short black sections subapically and a minute black mark at tip; spiracles small, orange-brown; body bearing a moderately dense ventro-lateral fringe of long, soft, white setae, remainder of dorsum sparsely covered with short, soft, pale setae, these more dense basally, nearly absent dorsally; and intermixed with sparse, stiff, long, dark setae spiracularly to dorsally.

Larvae bear a small, pale orange to pink-orange eversible neck gland, visible only upon eversion. Dorsally on A1, larvae bear a pair of silvery-colored fissures that represent the opening of the grooming glands. Caterpillars remain in a prepupal, non-feeding state for 2–3 days. During this time their ground color, including the head becomes a dingy yellow-green, highlighted with light-green (Figure 3b–c).

### Pupa (Figures 3d–f) n = 16; length = 17–19 mm; development time = 27–31 days

Overall shape boxy and rectangular, broadest at A3; hung upside down, strongly curled anterior to cremaster such that pupae is parallel to substrate; ground color bright light green with yellowish granulations, especially dorsally; dorsally adorned with conical protuberances on either side of midline on A3–A6, tip of each protuberance yellow infused with light brown; two smaller protuberances arising laterally at base of wingpads, and two arising laterally on A4; one large keel, slightly notched at the tip, arising mid-dorsally on T2 and projecting anteriorly over head, dorsal edge of this keel strongly marked with bright yellow; spiracles small and dark brown; terminal abdominal segments, immediately anterior to cremaster, bearing a ridge of small granulations, these creating a shelf-like ring across A10; cremaster same color as remainder of pupa, comprising brown hooks which attach to a dark brown silk pad.

### Larval behavior and parasitoids

Larvae generally consumed most of the molted cuticle from the previous instar, often leaving the caudal tails. When handled, they regurgitated a drop of clear green fluid, wiping this on the “attacking” fingers. First instars feed in loose aggregations on the same leaf, but in the second and subsequent instars, individuals disperse to adjacent leaves with the distance between larvae increasing with age. Older larvae were often found with 1–10 tachinid eggs glued to the surface of their head capsule and/or thoracic dorsum (n = 8, Figures 2c, 3h). Two observations of parasitoid attacks in the field were by tachinid flies; both were unsuccessful. In both instances, the fourth instar larvae were able to use the thrashing of their caudal tails, in conjunction with the long setae on the head, to effectively discourage the fly. By arching backwards, curling into a C shape, and thrashing their tails, they successfully dislodged the flies. Of 30 individual larvae of *A. adoptiva* collected and reared at YBS (3^rd^ through 5^th^ instars), five (16.7%) produced parasitoids. However, many caterpillars died before or during pupation, and these may have been parasitized as well. We obtained conclusive results for only 7 rearings, which indicated 74.1% parasitism. In all cases, parasitoid larvae emerged from the butterfly larva and pupated, but in most cases adult parasitoids failed to eclose. This may have been due to insufficient humidity once the remaining host plant leaves were removed from rearing bags.

**Figure 4.**
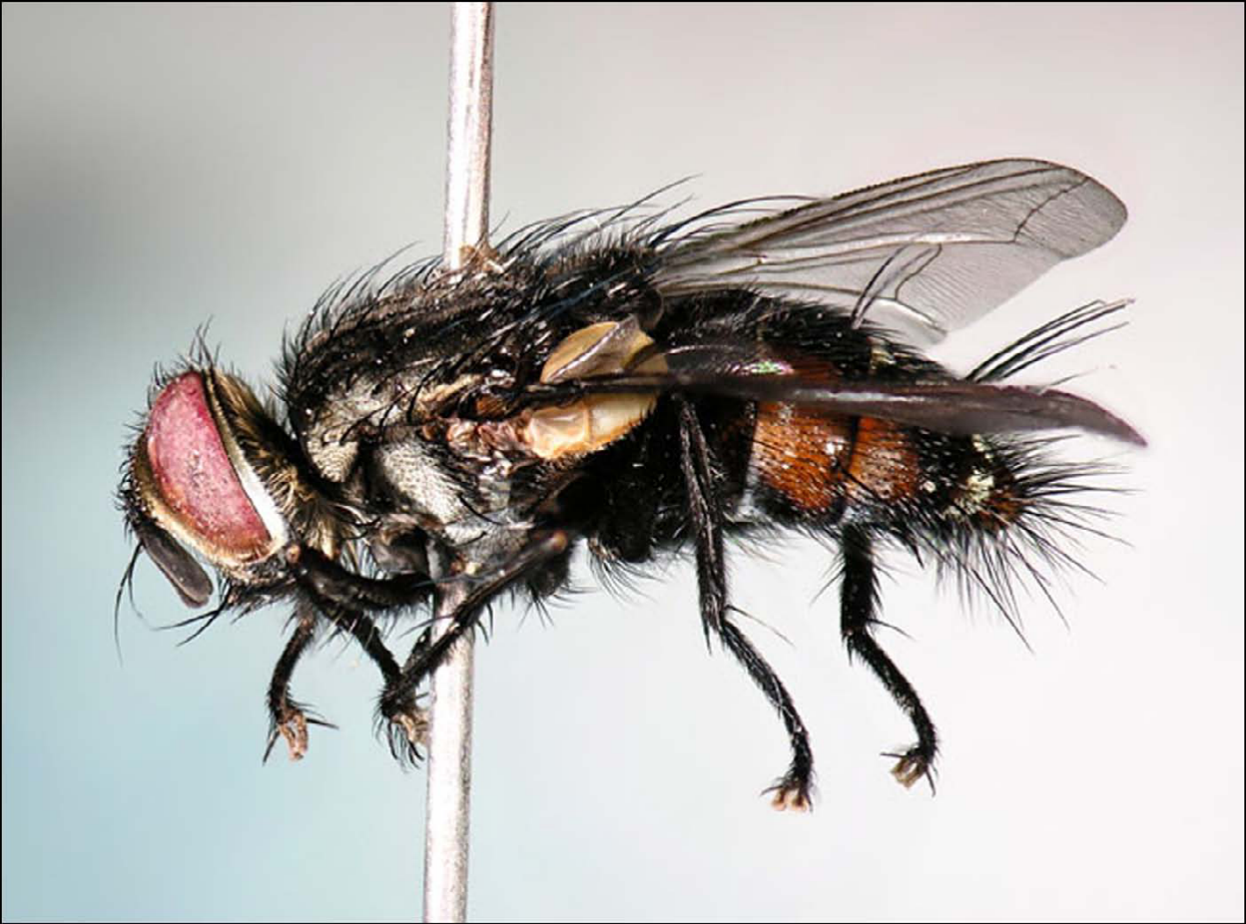
Adult *Winthemia* sp. reared from *Antirrhea adoptiva poiphyrosticta* in eastern Ecuador. Photo by Patrick McAfee.

We successfully reared a single adult tachinid and one “clutch” of approximately 30 braconids from *A. adoptiva*. The tachinid is a species of *Winthemia* Robineau-Desvoidy, 1830 (Figure 4), a widespread and species rich genus whose members attack a wide range of lepidopteran hosts (Wood 1987). Although our specimen keys to *W. analisella* Thompson, 1963, in Thompson's (1963) catalogue of the tachinids of Trinidad, it does not match the description precisely, and *W. analisella* has not been reported from Ecuador. It is highly likely that our specimen represents an undescribed species allied with *W. analisella*. The caterpillar from which this tachinid specimen was reared bore three conspicuous white eggs on its thorax/ abdomen. Such an arrangement is typical of *Winthemia*, where most species deposit large, hard-shelled eggs on the host cuticle, usually on the head and thorax (Wood 1987; JOS, unpublished obs.). Additional field observations of such parasitoid eggs on *A. adoptiva* larvae (and of parasitoid-host encounters, see above) suggest that parasitism by these tachinids may be frequent.

The braconid we reared is a newly described species (*Distatrix antireheae* Whitfield and Grinter; Grinter et al. 2009) in the subfamily Microgastrinae. In addition, two *A. adoptiva* larvae produced large, solitary hymenopteran parasitoids (probably Ichneumonidae) that pupated but failed to eclose. In one of these cases, the parasitoid larva formed a silken pupal chamber among leaf litter left in the rearing bag. These appear to be the first South American records of parasitism for *Antirrhea*. In Costa Rica, Janzen and Hallwachs (2007) reported two tachinids (*Anoxynops auratus* Thompson, 1968 and an unknown species) and one ichneumonid (species unknown) from *A. philocetetes lindigii* C. and R. Felder, 1862.

### Adult behavior

Adults of *A. adoptiva* (Figures 3g, 6) are crepuscular and generally active only at dusk in our study area. They are typically found within the forest or along forest edges, most often associated with stands of their food plant. Similar to the cloud forest inhabiting *A. pterocopha* from Costa Rica (DeVries 1987) and *A. weymen* from Colombia (Heredia and Alvarez-Lopez 2004), adults of *A. adoptiva* rest during the day under branches, tree trunks, or overhanging banks, flying only when disturbed. They are mainly active along streams. We have observed that males of *A. adoptiva* will guard a stand of *Chusquea* for up to six days, patrolling a linear area of up to 40 m and circling this plot approximately every five minutes. Males will chase nearly anything that flies into the area, including bats, small moths and larger crepuscular butterflies such as *Eryphanis greeneyi* Penz and DeVries, 2008 (Nymphalidae: Brassolini). Male flight is rapid and erratic, and they are difficult to observe in the hours of poor visibility during which they fly. Females show a slower, floppier flight pattern, generally resembling that of a large saturniid moth, a behavior consistent with that described for *A. pterocopha* (DeVries 1987). The paler redbrown coloration of the lower wing surface (Figure 6b) is distinctive in females and, in combination with their flight pattern, makes the sexes easily separable on the wing. We observed a single oviposition event; the female fluttered under bamboo stalks, bumping against the underside of the foliage several times. This was repeated at three different locations until she finally grasped a leaf, oriented herself along the mid-vein on the leaf's lower surface, and took approximately five minutes to lay a row of four eggs.

### Comparison with other species of *Antirrhea*

**Figure 5.**
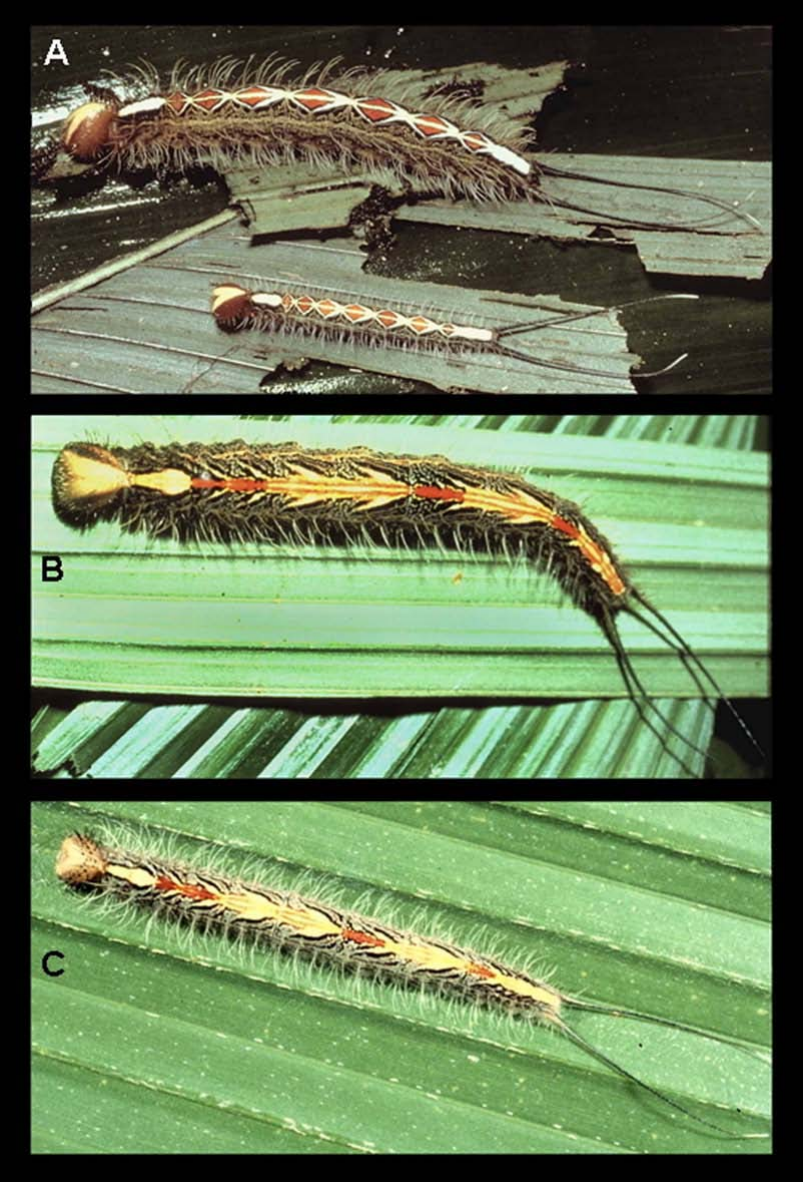
Mature larvae of *Antirrhea*; a) *A. pterocopha* Salvin and Godman, 1868, Costa Rica (PJD photo); b) *A. miltiades* (Fabricius, 1793), Costa Rica (PJD photo); c) *A. philoctetes* (Linnaeus, 1758), Trinidad (Hans Boos photo).

Color photographs of the fifth instars of four *Antinhea* species - *A. pterocopha* Salvin and Godman, 1868, *A. miltiades* (Fabricius, 1793), *A. philoctetes* (Linnaeus, 1758) (Figure 5) and *A. weymeri* (Heredia and Alvarez-Lopez 2004) - were available to us, allowing for comparisons with *A. adoptiva* (see Discussion for comments on nomenclature). An alcohol-preserved fifth instar of *A. pterocopha* was also available (PJD collection). The final instars of all species exhibit the same characteristic head capsule shape and unusual small scoli at the vertex as described here for *A. adoptiva*, but three different color patterns can be observed: (1) uniformly pale (*adoptiva, philoctetes*); (2) pale with a broad, dark brown frontal band and a large dark brown genal spot (*weymeri*); and (3) mostly brown with a large triangular pale marking anteriorly (*pterocopha, miltiades*). Body shape is relatively uniform, and all five species show bright, disruptive dorsal coloration, a moderately dense fringe of subspiracular setae, and elongate caudal tails. *Antirrhea pterocopha* and *A. miltiades* have a darker general appearance than the other three species. Although dorsal coloration patterns are species-specific, *A. adoptiva* and *A. weymen* have a longitudinal, pale yellow stripe flanking the central pattern that is absent in the other three. The dorsal patterns of *A. miltiades* and *A. philoctetes* are similar in showing sequential chevrons interspersed by red markings. *Antinhea pterocopha* departs from the other four species in having a row of white, diamondshaped markings that enclose paired red triangles. The relative lengths of the caudal tails and body vary across species; in *A. pterocopha* and *A. philoctetes* caudal length is approximately 50 % of the body length, it is roughly 30 % in *A. miltiades*, and less than 30% in *A. adoptiva*. Interestingly, the caudal tails in first through fourth instars of *A. adoptiva* were proportionately longer than those of the fifth instar (estimated from the longest body length before each molt as listed in the description above). Finally, the paired, dorsal grooming glands on A1, described for *A. weymen* by Heredia and Alvarez-Lopez (2004), also occur in *A. adoptiva* and *A. pterocopha*. These may be present throughout the genus. It is clear that color pattern elements are shared by various species of *Antirrhea*, and these may prove useful in future systematic studies.

## Discussion

The early stages of *A. adoptiva* described here are similar to those described for other *Antinhea* species (Heredia and Alvarez-Lopez 2004, DeVries 1987, Figure 5). The major difference in coloration, apparent in advanced instars, is the lack of distinct markings on the anterior portion of the head (also in *philoctetes*). Otherwise, judging from published life histories and available photographs, the larvae and pupae of *A. adoptiva* fit the gestalt of *Antinhea*. A major novelty, however, is that this is the first record of an *Antirrhea* feeding on bamboo (Poaceae) (Ackery 1988). All previous records are on palms (Arecaceae) (DeVries et al. 1985; DeVries 1986, 1987; Urich and Emmel 1990; Matos 2000; Heredia and Alvarez-Lopez 2004). Two additional morphine genera, *Caerois* Hübner and *Morpho* Fabricius, are known to have host plant associations with the grass family (Poaceae) (DeVries et al. 1985; DeVries 1987). However, until more life histories become available for the Morphinae, the importance of the data presented here cannot be fully evaluated.

It has been established that larval morphology and specificity of parasitoids can be valuable for species recognition in groups where adults cannot be easily separated (e.g., Francini et al. 2004; Hebert et al. 2004). Therefore, life history studies contribute important traits for characterizing species, and this in turn may have implications for recent nomenclatural changes proposed within *Antirrhea*. For example, in most butterfly literature during the past 80 years, *A. philoctetes lindigii* was known as *A. miltiades* (Fabricius, 1793), a taxon only recently synonymized due to a nomenclatural technicality (Lamas 2004). Here, we note that, in addition to obvious differences in dorsal HW coloration between Central and South American specimens of *A. philoctetes* (*sensu lato*), there are distinct differences in larval coloration as well: caterpillars of Costa Rican *A. philoctetes lindigii* and Trinidadian *A. philoctetes philoctetes* are easily separated based on head and body color patterns (see DeVries *et. al*. 1984; Urich and Emmel 1990; and Figure 5). This, of course, raises the question of whether Central and South American “*philoctetes*” in fact represent a single species. Perhaps more detailed studies of larvae, adults and parasitoids of *A. philoctetes* will help resolve the taxonomic question raised here. We predict that larval stages will be extremely important for elucidating species concepts in *Antinhea*, and perhaps throughout the Morphinae.

### Editor's note

Paper copies of this article will be deposited in the following libraries. Senckenberg Library, Frankfurt Germany; National Museum of Natural History, Paris, France; Field Museum of Natural History, Chicago, Illinois USA; the University of Wisconsin, Madison, USA; the University of Arizona, Tucson, Arizona USA; Smithsonian Institution Libraries, Washington D.C. USA; The Linnean Society, London, England.

**Figure 6.**
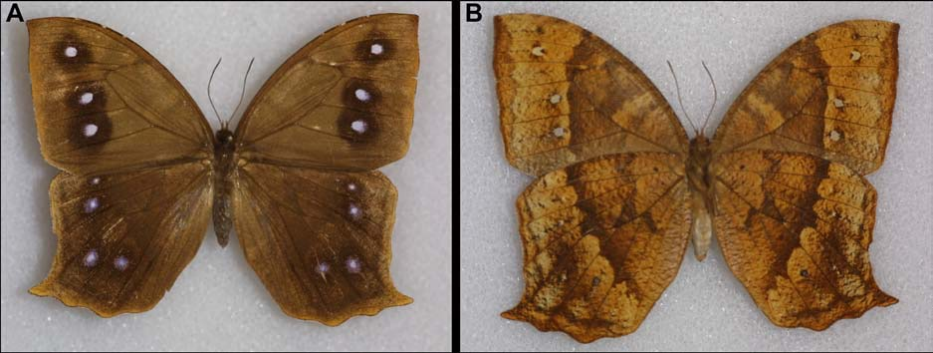
Adult female of *Antirrhea adoptive porphyrosticta*; a) dorsal view; b) ventral view.
